# Investigating metal distribution patterns in pristine and ocean-weathered plastics using LA-ICP-TOFMS

**DOI:** 10.1039/d5ja00223k

**Published:** 2025-09-08

**Authors:** Lyndsey Hendriks, Matthias Egger, Denise M. Mitrano

**Affiliations:** a Institute of Analytical Chemistry, University of Vienna Währingerstraße 38 1090 Vienna Austria lyndsey.hendriks@univie.ac.at; b The Ocean Cleanup Coolsingel 6 3011 AD Rotterdam The Netherlands; c Empaqtify Ullmannstrasse 13a 9014 St. Gallen Switzerland; d Department of Environmental Systems Science, ETH Zurich Universitätstrasse 16 8092 Zurich Switzerland

## Abstract

Plastic pollution in marine environments poses ecological risks, in part because plastic debris can release hazardous substances, such as metal-based additives. While microplastics have received considerable attention as vectors of contaminants, less is known about larger macroplastics and their role in the spatial and temporal redistribution of substances. In this study, pristine, store-bought plastic items and macroplastics recovered from the North Pacific Subtropical Gyre (NPSG) were analysed using Fourier-Transform Infrared Spectroscopy (FTIR) to identify polymer types, and bulk acid digestion followed by Inductively Coupled Plasma Mass Spectrometry (ICP-MS) for total metal quantification. These techniques were complemented by high resolution elemental mapping by Laser Ablation Inductively Coupled Plasma Time-of-Flight Mass Spectrometry (LA-ICP-TOFMS). Detailed elemental maps revealed native metal distribution in pristine plastics, and evidence of both sorption and intrinsic metal depletion in weathered plastics. In particular, weathered plastics showed surface depletion of intrinsic metals, and enrichment of seawater-derived elements (*e.g.*, Na, Mg, I). Linear regressions were used to quantify spatial distribution trends across cross sections, providing statistical support for directional gradients. Since pristine and weathered plastics were opportunistically collected, variability in product type, polymer chemistry, and weathering time limited direct comparisons. Instead, this study demonstrates the utility of LA-ICP-TOFMS for mapping elemental distribution in plastics, offering a novel analytical approach for investigating spatial metal distribution in plastics and laying the groundwork for future studies on weathering processes in marine environments.

## Abstract 2

LA-ICP-TOFMS mapping reveals metal distribution patterns in ocean-weathered plastics, providing new insights into leaching and sorption processes following real-world environmental exposure.

## Graphical abstract

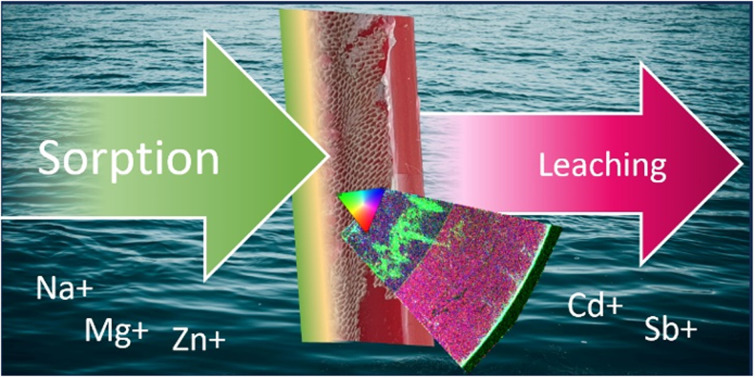

## 1. Introduction

Plastic pollution is a persistent environmental issue, with significant amounts of plastic waste entering the environment, including oceans.^1–4^ Beyond visible accumulation^5,6^ and negative environmental impacts of this waste,^7–9^ an additional consideration is the chemical and physical transformations plastics undergo upon weathering.^10^ The persistence of plastics in the ocean can pose several ecological risks, as they can potentially: (1) adsorb and concentrate hazardous substances, acting as vectors for contaminants and (2) leach chemical and metal additives, potentially releasing harmful elements into the environment.^11–14^ Metals are routinely incorporated as stabilizers, pigments, or catalysts during manufacturing, and metals concentrations and composition can vary widely depending on the material.^11^ Environmental exposure, including processes such as UV-induced degradation, leaching, and adsorption, can alter both the surface and internal composition of plastics over time. The rate and extent of leaching may vary depending both on the plastic properties and type and degree of weathering. Additionally, plastics in the ocean are often colonized by marine organisms (referred to as biofouling),^15^ which may ultimately influence metal sorption, release, and weathering patterns.^14,16^ While some studies have explored sorption under specific conditions—often influenced by pH, surface chemistry, and metal availability—several have shown that leaching from embedded additives (*e.g.*, pigments, stabilizers) may represent a substantial pathway for metal release into the environment, especially following weathering.^17–19^

Understanding the interactions between plastics, their additives, and marine environments necessitates advanced and robust analytical tools. While techniques such as Fourier-Transform Infrared spectroscopy (FTIR) and Raman spectroscopy provide valuable insights into polymer composition and weathering processes,^20^ these techniques cannot detect the incorporation, absorption or leaching of inorganic species contained within the plastic. Understanding the spatial distribution of metals within plastic—both in pristine and weathered states—is critical for evaluating metal-plastics dynamics. A variety of techniques are available to detect metals on material surfaces, including Scanning Electron Microscopy (SEM), Energy Dispersive X-ray Spectroscopy (EDX), X-ray Photoelectron Spectroscopy (XPS) and Laser Ablation Inductively Coupled Plasma Mass Spectrometry (LA-ICP-MS).^21,22^ Historically applied in materials sciences for characterizing coatings on metals and in earth sciences for studying isotopic variations and trace element zoning in minerals,^23^ LA-ICP-MS has recently been adopted for analysing weathered microplastics (MPs).^24–26^ LA-ICP-MS has successfully been used to generate metal concentration profiles in MPs collected from marine systems by analysing their subsurface layers (∼200 μm).^24^ Their study provided critical insight into the distinction between sorbed and intrinsic metal distributions, but was limited to near-surface regions. Building on this, a subsequent study advanced this approach by combining LA-ICP-MS and Laser Induced Breakdown Spectroscopy (LIBS) to study biofilm covered MPs in controlled freshwater and wastewater settings.^26^ Their results confirmed that laser-based techniques can successfully be applied to monitor both leaching and adsorption processes, although the focus remained on smaller MPs and controlled ageing. While MPs have garnered significant attention due to their small size and widespread distribution, macroplastics, offer distinct analytical advantages due to their larger physical dimensions. These include easier handling, and the possibility to obtain cross-sections for internal *versus* external regions of interest. This makes them particularly suitable for studying spatially resolved metal distributions, including potential leaching of intrinsic additives or sorption of environmental contaminants. Although it remains uncertain how these patterns translate to MPs, macroplastic items provide an important opportunity to examine early-stage weathering processes and the redistribution of metals within the plastic matrix.

Thus, building upon previous work, here we present a novel application of Laser Ablation Inductively Coupled Plasma Time-of-Flight Mass Spectrometry (LA-ICP-TOFMS) to analyse pristine plastics and naturally weathered plastic items collected from the North Pacific Garbage Patch (NPGP).^5,27^ A focused laser beam is used to raster across the sample surface, ablating material that is then transported to the ICP-TOFMS for analysis. Each shot corresponds to an individual pixel in the resulting map, allowing LA-ICP-TOMFS to generate high-resolution elemental maps of solid samples.^28^ A key advantage of using a TOFMS-detector lies in its ability to simultaneously detect the full suite of elements across a broad mass range (*m*/*z* 14 to 254),^29,30^ which provides a complete and comprehensive picture of the metals embedded and associated with the plastic samples. Comparing environmental weathered plastic items with new, store-bought, counterparts, provides a deeper understanding of how the metals distribution in the plastic could change due to exposure to sunlight, seawater, and mechanical stress in marine environments. The recorded elemental patterns revealed localized enrichments, depletion zones, and directional gradients—patterns that offer insights into the extent of metal leaching over time. Consequently, this proof-of-concept study takes a step forward in providing high-resolution elemental maps to reveal spatial patterns that emerge after environmental exposure.

## 2. Materials and methods

### 2.1. Sample collection

**Fig. 1:**
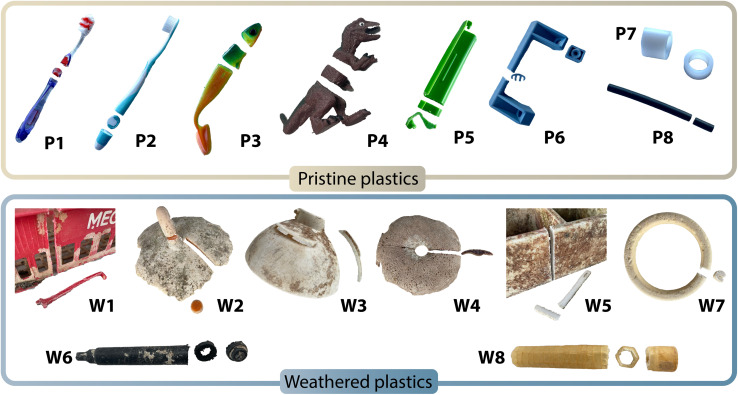
Overview of the collected samples and their respective cuts for cross-sectional analysis.

A total of 16 plastic items representing a diverse suite of products and material chemistries were collected and analysed. These include 8 pristine plastics and 8 ocean-weathered plastics. An overview of the different items is presented in Fig. 1.

Pristine plastics (P1–P8) were purchased from local stores in Switzerland in 2024, and were used to assess the variability and homogeneity of metal distribution as initially produced. This included two toothbrushes, a plastic handle, a plastic dinosaur toy, a vertical bouncer fish as well as common laboratory items such as PTFE tubing.

Ocean-weathered plastics (W1–W8), which were weathered under natural conditions for unknown amounts of time, were collected by The Ocean Cleanup (see Fig. S1 for an overview). These samples were recovered from the NPGP, an open ocean plastic accumulation area located between California and Hawaii, between 2015 and 2018. Plastics afloat in these offshore waters have been shown to often be decades old.^31,32^ A detail description of the sampling area and procedure can be found elsewhere.^27^

While the pristine and weathered plastics were opportunistically collected and thus differed in product type and chemistry, our goal was to use the pristine samples as a baseline for native metal distribution, allowing for comparison with the weathered counterparts. Metal content and distributions vary with product manufacturing, thus our results illustrate general patterns rather than absolute concentrations across all plastics.

### 2.2. Reagents and materials

All reagents used were of high purity. Concentrated nitric acid (HNO_3_, 69%, Rotipuran Supra, Carl Roth, Karlsruhe, Germany) and hydrogen peroxide (H_2_O_2_, 31%, ultrapure, Merck, Darmstadt, Germany) were employed for acid digestion. Calibration standards were prepared gravimetrically using ICP-MS multi-element and single-element standards (Quality Control Standard, 48 elements; LabKings, Hilversum, The Netherlands), covering a concentration range of 0.001–10 μg L^−1^. Ultrapure water (18.2 MΩ cm, ELGA PURELAB Ultra MK2, Veolia, Lane End, UK) was used throughout for dilutions and sample preparation.

### 2.3. Sample preparation

#### 2.3.1. Sectioning for cross-section analysis

To assess differences in metal distribution between the surface and interior of the plastics, all samples – both pristine and ocean-weathered – were sectioned to expose full cross-sections (Table S1). Cutting locations were selected to capture both outer surfaces and the inner bulk material. Each cross-section thus spanned from one exterior surface through the core to the opposite surface. For weathered plastics, the outer surfaces had direct content with environmental media (*i.e.*, seawater), whereas the pristine plastics did not. This approach enabled a direct comparison of metal accumulation on the surface—associated with sorption from seawater or biofilms—and internal gradients indicative of additive diffusion or degradation processes. After cutting the samples into centimetre-sized pieces with a hand saw, they were polished using Silicon Carbine sanding paper of progressively finer grain sizes (P80 to P1000). Polishing is a standard step in LA-ICP-MS to ensure a smooth, uniform surface for consistent ablation and signal quality. While this process removes a thin outer layer of material, the sanding is not expected to significantly alter the bulk metal distribution within the plastic or induce cross-contamination between different regions of the sample. The samples were then rinsed with MilliQ water to remove unbound particles and dust before analysis.

#### 2.3.2. Sample digestion for total metal quantification

To quantify the total metal content in each plastic item, an acid digestion^33–35^ followed by ICP-MS analysis was performed on the different plastic items. Approximately 200–400 mg of plastic was cut and digested in a 3 : 1 v/v mixture of concentrated nitric acid (Rotipuran HNO_3_ supra 69%, Roth, Karlsruhe, Germany), and hydrogen peroxide (H_2_O_2_ ultrapure 31%, Merck, Darmstadt, Germany) using a closed-vessel microwave digestion system (Multiwave Pro, Rotor 24HVT50, Anton Paar, Graz, Austria). PTFE vials were used, thus items P7 and P8 were unfortunately not digested as these consisted of PTFE. Digested samples were subsequently diluted to a final volume of 10 ml with ultrapure water (ELGA PURELAB Ultra MK2 Water Purification System, resistivity: 18 MΩ cm, ELGA LabWater (Veolia Water Technologies), Lane End, United Kingdom) and filtered as needed.

### 2.4. Plastic characterization by spectroscopic and elemental analysis

To complement the elemental mapping and better understand the materials analysed, different analytical techniques were applied to characterize the plastic samples. These included microscopy for surface morphological inspection, FTIR spectroscopy for polymer identification and bulk microwave digestion followed by ICP-MS for total metals quantification. For multi-material items such as toothbrushes (items P1 and P2), only the rigid hard plastic components were analysed. The soft, rubber-like sections were excluded from the analysis.

#### 2.4.1. Optical microscopy

To visually assess the degree of physical weathering and surface degradation, selected plastic samples were examined under an optical microscope (Axioscope 7, Carl Zeiss Microscopy Deutschland GmbH, Oberkochen, Germany). This was done to assess surface features such as cracks, abrasion, or other signs of degradation. These alterations could potentially be related to UV-weathering or mechanical stress. In one case (item W1), the surface exhibited a mesh-like structure consistent with biological colonization, possibly from marine organisms. Such features may influence surface chemistry and extent of metal leaching and were documented to support interpretation of subsequent elemental mapping.

#### 2.4.2. FTIR spectroscopy

Polymer composition was determined using an FTIR spectrometer (Tensor 37, with a platinum ATR module and a diamond ATR crystal, from Bruker Optik GmbH, Ettlingen, Germany), operating in the range of 4000–400 cm^−1^ with a resolution of 4 cm^−1^. Each measurement consisted of 32 scans, averaged to improve signal-to-noise ratio. The plastic cross-section prepared for LA-ICP-TOFMS were measured in their centre to avoid weathered areas with potential interferences. Resulting spectra were matched against a reference polymer library using the software (OPUS v.8.2.28) and the built-in database of the instrument.

#### 2.4.3. ICP-QQQ-MS

Analysis of the digested samples was performed using a Triple Quadrupole ICP-MS (ICP-QQQ-MS, Agilent 8800, Agilent Technologies, Tokyo, Japan). The ICP-MS instrument was equipped with an ASX 500 autosampler (Agilent Technologies, Waldbronn, Germany) and was tuned in no gas, He KED and O_2_ gas modes for highest sensitivity (full operating parameters can be found in Table S2). A total of 49 elements were quantified, and for each, the isotope with the highest natural abundance and lowest potential interferences was selected. Elemental concentrations were determined using the Agilent MassHunter software package (Workstation Software, Version C. 01.06.2019). Internal standards (Ge and Rh) were added online to correct for matrix effects and signal drift.

### 2.5. Elemental mapping by LA-ICP-TOFMS

A 193 nm excimer laser ablation system (ImageGEO 193, Elemental Scientific Lasers, Bozeman, MT, USA), equipped with a two volume (TwoVol3, ESL) ablation cell was coupled to an ICP-TOFMS (icpTOF 2R, TOFWERK, Switzerland) *via* a dual concentric injector (DCI, ESL). Using TOFPilot (TOFWERK, Switzerland) to interface with the laser system ActiveView2 (AV2, ESL), this setup enabled the execution of ablation patterns with per-shot synchronization, including laser metadata, automatic sensitivity tuning, and a live preview of the ablated images. The LA-ICP-TOFMS conditions were optimized using a NIST 612 glass material to maximize sensitivity, minimize oxides and to obtain adequate signal washout. The full set of operating conditions can be found in Table S3.

On each plastic cross section, a lasso-pattern, which allowed to flexibly outline irregularly shaped samples, was set up before being scanned by the laser. The analysis of a greater proportion of the sample is expected to be more representative of the average value of the elemental intensity compared to focusing on individual spots or lines. The specific ablated areas used for analysis are highlighted in blue in Table S1.

### 2.6. Data processing and statistical analysis

The recorded datafiles were first analysed using an open-source screening software – TOFhunter^36^ – to identify analytes presents. Subsequently, the data were imported into a data visualization and processing software – Iolite^37,38^ (v 4.9.4, ESL, Bozeman, MT, USA) – to produce the elemental maps and cross-section profiles. For data interpretation and presentation, isotopes were selected based on their highest natural abundance and minimal spectral interferences. While TOFMS allows the collection of a full mass spectrum (*m*/*z* 14 to 254),^29,30^ only the most abundant or interference-free isotopes are shown in the figures for clarity. Python 3.8.0 was used for further calculations and plotting. Due to the lack of an appropriate matrix-matched standard for the plastic materials, absolute quantification of elemental concentrations was not performed. Thus, to avoid misinterpretation arising from differences in isotopic abundance, spectral interferences, and element-specific sensitivity (*e.g.*, ionization efficiency, transmission, and ablation yield), for each element the signal intensity was normalized to its maximum value within the analysed area, resulting in relative intensity maps ranging from 0 to 1.

Additionally, to evaluate whether the observed elemental profiles reflect meaningful directional trends or random variation, we applied simple linear regression fits to the central portion of each normalized intensity profile, excluding the rise and tail regions to minimize edge-related effects. Three metrics were extracted from each regression: the slope, the coefficient of determination (*R*^2^) and the *p*-value for the slope. The *R*^2^-value quantifies the goodness of fit to a linear model; values closer to 1 indicate a stronger correlation. The *p*-value assesses whether the observed slope differs significantly from zero. A *p*-value < 0.05 is considered statistically significant, supporting the observation of an actual gradient rather than random variation.

## 3. Results and discussion

### 3.1. Material characterization

FTIR analysis revealed that the pristine samples included a broader range of materials, including polypropylene (PP), polyethylene terephthalate (PET), polystyrene (PS), polytetrafluoroethylene (PTFE), and diisooctyl phthalate. On the other hand, the weathered plastic samples were predominantly composed of polyethylene (PE) and polypropylene (PP). A summary of the assigned polymer composition is provided in Table S4. Although the polymer compositions do not directly match between pristine and weathered samples, and the absence of PE among the pristine samples limits a one-to-one comparison, the pristine materials still serve as a useful baseline for understanding typical metal content and distribution prior to environmental exposure. Indeed, similarities in metal content between polymer types have been demonstrated in previous studies.^35^

The metal content quantification by bulk ICP-MS revealed the presence of various metals in both pristine and weathered samples, with varying concentrations ranging from μg kg^−1^ to mg kg^−1^ (see Fig. S2, and Table S5 for LOQs). Different metals are commonly used as additives or stabilizers in plastic production,^11^ which explains their presence in these samples. Predominant analytes observed were Na, Mg, Al, Ca, Ti, Fe, Zn, Ba and Pb. Sodium sulfate (NaSO_4_), magnesium silicates – also known as talk, calcium carbonate (CaCO_3_), zinc oxides and barium sulfate (BaSO_4_) are common plastic filler materials and stabilizers while Al, Ti and Pb are used as inorganic pigments, which explains their high abundances.^11^ While the original plastic manufacturing recipe is unknown for each item, the recorded values are within the same range as reported values from previous studies investigating metal content in plastics.^11,35,39^ Item P6, a blue plastic handle, exhibited elevated concentrations of Na, Al and Cr in the mg kg^−1^ range. Given its distinct blue colour, these elements are likely associated with the presence of blue pigments such as ultramarine blue—a sulfur-containing sodium aluminosilicate—and potential chromium-based compounds, which are occasionally used to modify colour tone or arise from recycled pigment mixtures. Interestingly, trace levels of rare earth elements (REE), comprising the fifteen lanthanide elements (La through to Lu, plus Y and Sc) were detected in items P2 and P6, suggesting that these could be recycled plastics.^40^ Weathered samples generally exhibited a slight increase in the concentration of Se and Cd indicating that the weathering process may lead to the sorption of certain metals from the surrounding environment. However, it should be noted that a clear distinction between sorbed and embedded elements is not possible from this bulk information alone.

For example, item W2, which was identified as an orange buoy, contained a much higher Cd concentration (85.2 mg kg^−1^) than all other plastics analysed in this survey. This suggests that Cd was likely added during manufacturing, possibly as a pigment (cadmium orange),^13,41^ which is supported by the co-detection of Mo, also an orange pigment. However, since the original composition of these weathered plastics is unknown, it remains difficult to determine whether Cd was originally incorporated during production, later adsorbed from the environment, or influenced by both processes. This ambiguity illustrates a key limitation of bulk ICP-MS: while it provides total metal concentrations in plastic samples, it does not distinguish whether metals are incorporated within the polymer matrix, sorbed onto the surface, or associated with biofilms. This limitation makes it difficult to interpret the impacts of environmental weathering on the distribution of metals detected or how these metals diffuse through plastic. To address this gap, spatially resolved techniques such as LA-ICP-TOFMS are needed to differentiate between surface-enriched (potentially sorbed) elements and those distributed throughout the polymer matrix (likely intrinsic). Spatially resolved mapping offers insights into metals distribution patterns and potential mobility during environmental weathering.

Furthermore, it should be noted that while the detection of these metals aligns with common plastic manufacturing processes and expected metals from exposure, the varied origins and unknown initial conditions of the samples limit further interpretation. Therefore, any observed differences—or similarities—in elemental concentrations between pristine and weathered samples cannot be directly interpreted as indicative of metal stability within the plastics during weathering. Rather, they should be viewed in the context of the inherent variability in both initial composition and environmental exposure history. Establishing this baseline material characterization is essential for interpreting the results of subsequent LA-ICP-TOFMS analysis.

### 3.2. Analysis of pristine plastic

**Fig. 2:**
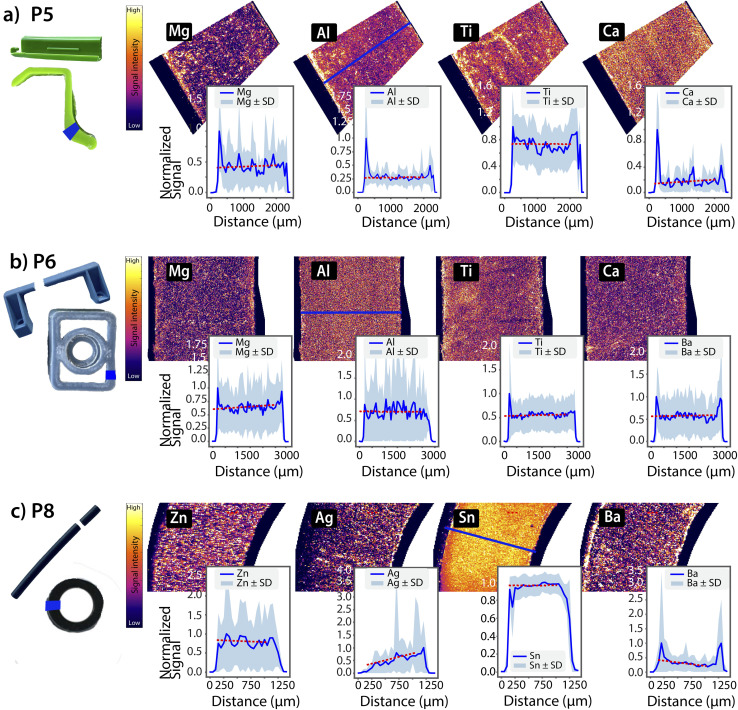
False-colour elemental distribution maps with overlaid cross-sectional intensity profiles (blue) for selected pristine items: (a) P5, (b) P6, and (c) P8. Red dashed lines represent linear regressions fits to the central region of each profile to help visualize trends. While no consistent gradients are apparent, the profiles reveal sporadic spikes and irregularities, indicative of localized inhomogeneities in additive distribution within an otherwise homogeneous matrix.

Pristine samples presented smooth surfaces with no visible signs of degradation, micro-cracks or other surface irregularities. Upon cross-sectioning, no colour gradients were observed. Cross-section elemental maps and profiles were generated to assess the distribution of elements across the plastic samples. Selected examples are presented in Fig. 2. While no consistent gradients were observed, the profiles revealed sporadic spikes and irregularities, indicating localized inhomogeneities. These observations align with recent studies using LA-ICP-MS, which have highlighted the non-uniform distribution of additives and fillers in plastic matrices.^42,43^ These localized hotspots likely result from the manufacturing process, such as uneven dispersion of pigments, fillers, or catalysts, rather than post-production transformation. Edge-enriched signals observed in some samples further support this interpretation and may stem from surface treatments, additive migration during extrusion, or other production-related processes.^44,45^

To assess the presence of directional trends in elemental profiles, linear regressions were applied to the central portions of normalized intensity profiles. A summary of regression results is provided in Table 1. The majority of the elemental profiles were flat (slope ≈ 0, *R*^2^ < 0.2, *p* > 0.5), indicating no directional changes. Overall, minimal or non-significant trends were observed for the pristine samples, supporting the interpretation of an overall homogeneous additive distribution with isolated microscale heterogeneities.

**Table 1:** Summary of linear regression statistics (*R*^2^-value, *p*-value and % change in signal intensity) for normalized cross-sectional intensity profiles, recorded in pristine plastics from Fig. 2. Elements with high *R*^2^ and low *p*-values (*e.g.*, Ag) show a clear spatial gradient, whereas others (*e.g.*, Mg, Al, Ti) do not exhibit statistically significant variations across the profiles

	Element	*R* ^2^-value	*p*-value	% Change
P5	Mg	0.00	0.39	—
	Al	0.00	0.71	—
	Ti	0.00	0.97	—
	Ca	0.00	0.12	—
P6	Mg	0.01	0.02	—
	Al	0.00	0.92	—
	Ti	0.00	0.17	—
	Ba	0.00	0.57	—
P8	Zn	0.00	0.56	—
	Ag	0.54	2.67 × 10^−6^	−27%
	Sn	0.00	0.89	—
	Ba	0.38	6.88 × 10^−5^	—

**Fig. 3:**
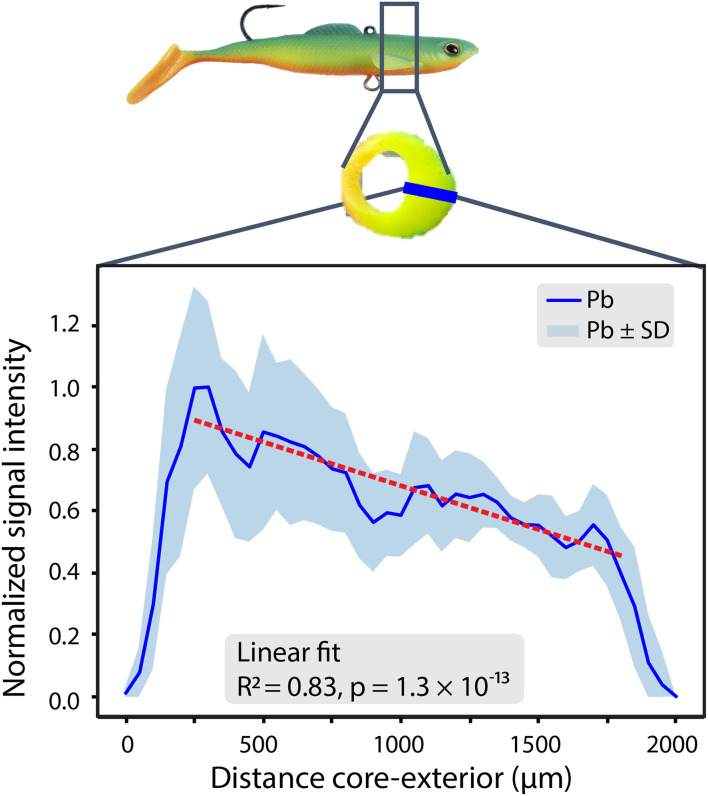
Pb profile measured in item P3, displaying an increased Pb signal near the metal contact area (core) and a decline moving away from it (exterior), which supports the hypothesis of localized transfer from the jig head.

However, two exceptions were identified among the pristine samples. First, in sample P8 (Tygon® tubing), Ag exhibited a pronounced and statistically significant outward-to-inward gradient (*R*^2^ = 0.74, *p* = 2.7 × 10^−6^). The Ag concentration was highest at the interior surface and declined toward the outer edge of the tubing, consistent with the intentional incorporation of Ag-based antimicrobials during production. No similar gradients were observed for other elements in P8, supporting their role as bulk fillers rather than surface-active agents. Second, in sample P3, a fishing bouncer, a clear Pb gradient was observed (Fig. 3). During the sample preparation, a jig head embedded in the plastic was identified as the likely source, being coated with lead or a lead-containing component. This direct contact resulted in elevated lead concentrations (60 mg kg^−1^), likely due to the transfer of Pb from the jig head into the plastic over time. A linear regression was applied on the central portion of the Pb intensity profile (edge regions were excluded) and revealed a strong linear correlation (*R*^2^ = 0.83) with a significant *p*-value (*p* = 1.3 × 10^−13^), confirming that the Pb decrease across the profile was statistically significant and is unlikely due to random variation. Based on this fit, a 48% decrease in normalized signal intensity was determined. This change indicates relative metal leaching or redistribution within the material. However, due to the absence of matrix-matched calibration, such changes remain semi-quantitative and should be interpreted as within-sample proportional changes rather than absolute concentration changes. The accumulation of Pb on the side of the plastic in contact with the pristine jig head underscores the inherent ability of plastics to uptake or retain substances from their immediate surroundings.

Together, these results demonstrate that pristine plastic items generally lack progressive elemental gradients. Instead, they display isolated microscale inhomogeneities or surface enrichments that can be attributed to manufacturing processes, such as uneven additive distribution or surface treatments. These pristine samples serve as a baseline for typical metal distribution profiles in plastics which can be used to contrast how environmental weathering may alter metal distribution patterns across the material.

### 3.3. Analysis of weathered plastic

**Fig. 4:**
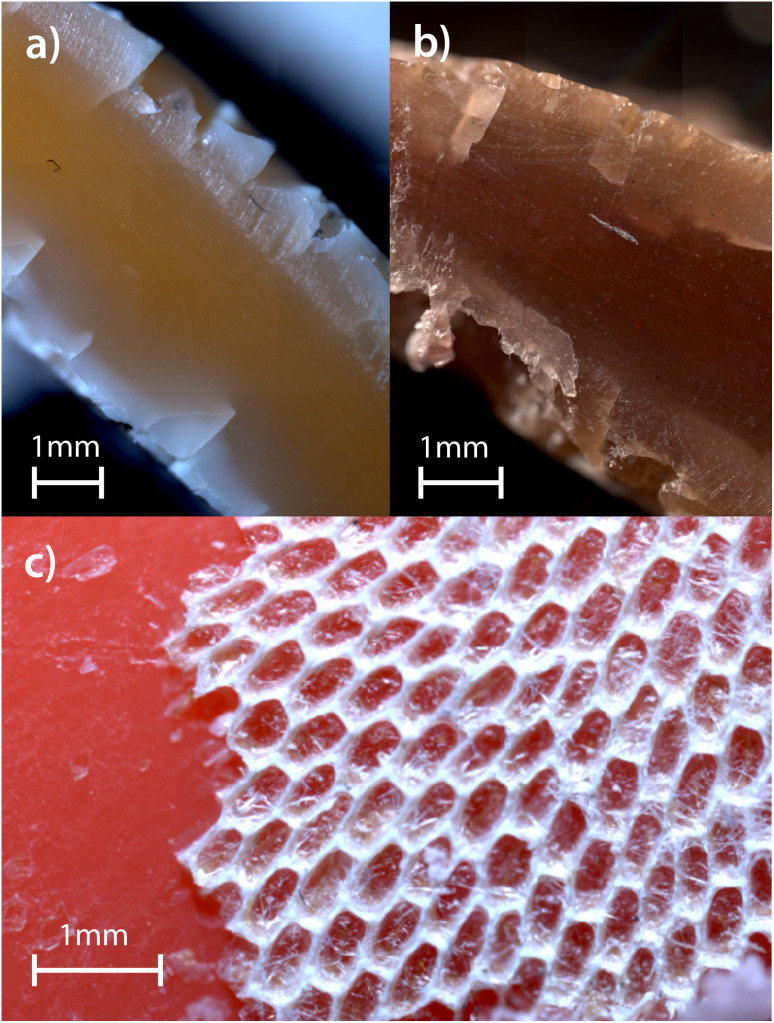
(a) and (b) Cross-sectional microscopy images of weathered plastic items W2 and W4, respectively. Both exhibit signs of degradation, including discoloration, and surface cracking. (c) Surface microscopy image of weathered plastic item W1, showing a smooth surface and the presence of biofouling.

Although the origin and duration of exposure for the specific items collected from the NPGP are unknown, it is likely that individual items experienced different durations of exposure and weathering conditions. During sample preparation (*i.e.* cutting of the plastic items), a qualitative assessment of the mechanical properties such as hardness and brittleness was made by whether it was easy or not to cut the plastic. Visual inspection of exposed cross-sections provided additional information about internal structures and surface changes in the weathered plastics. Across the eight weathered plastics items (W1–W8), notable variations in surface texture, colour and brittleness were observed (see Table S6). Some samples displayed rough, fractured surfaces—features commonly associated with prolonged weathering—while others appeared relatively intact. Microscopy images of samples W1, W2, and W4 (Fig. 4) further illustrate the extent of surface roughness, cracking, and discoloration. These visual changes likely reflect a combination of degradation processes such as UV-induced oxidation and mechanical fragmentation.^46–48^ For example, colour fading and embrittlement are consistent with UV-induced oxidation, while surface cracking and fragmentation may result from mechanical stress. Signs of biological colonization were also present, most clearly in sample W1, where visible biofouling was observed on the surface (Fig. 4c).

**Fig. 5:**
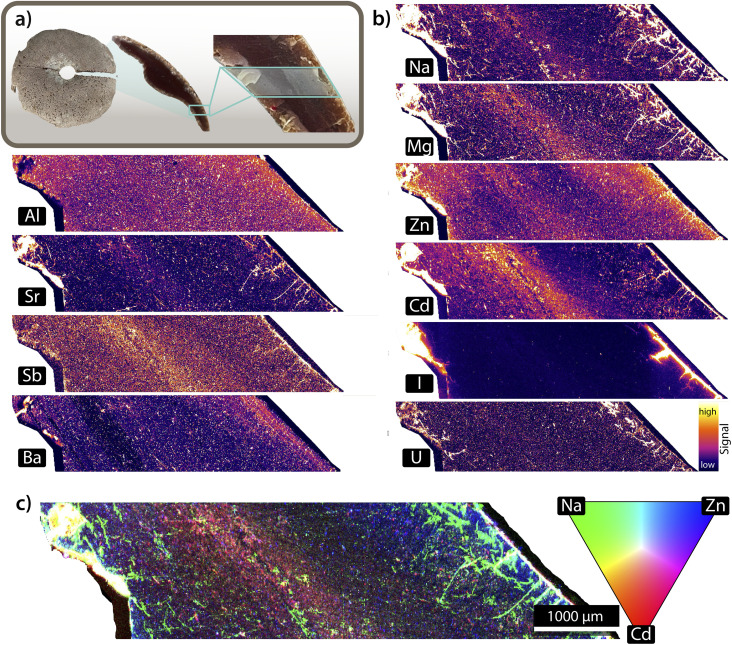
(a) Presentation of item W4, with a zoom-in on the ablated area. (b) Selected single elemental maps revealing different elemental distribution patterns. Sorption patterns: elements such as Na, Mg, Sr, I and U appeared to accumulate along cracks and weathered rims. (b) Leaching patterns: elements such as Al, Zn, Cd and Sb presented internal gradients, with higher intensities within the plastic matrix compared to areas where the plastic was in direct contact with seawater. (c) False-colour RGB composite image, obtained by combining three individual elemental maps with Na (green), infiltrating through the surface cracks, Cd (red) presenting an internal gradient and Zn (blue) illustrating both sorption and leaching.

To better understand metal distributions in marine-weathered plastics, we generated a set of elemental maps (Fig. 5) focusing on two groups of elements: (1) those commonly present in seawater, which may sorb onto plastic surfaces, and (2) those likely embedded in the polymer matrix, which may exhibit leaching or internal re-distribution. Item W4, a brown buoy, was selected as an example here, as microscopic examination revealed multiple fractures within the material (Fig. 4), and its brittle nature, noted during sample preparation, both suggested advanced weathering. When comparing elemental maps with structural features, seawater-derived elements (*e.g.* Na, Mg, Sr, and I) showed a clear enrichment at the surface of the plastic, as well as along cracks and fractures, which often aligned with physical changes, suggesting a strong correlation between physical and chemical weathering processes. While cracks varied in sizes within one plastic item and across materials, the largest crack measured was approximately 1.4 mm in depth. These cracks offer potential pathways for deeper penetration of seawater-derived elements into the plastic matrix. As Na, Mg, Sr, and I are abundant in marine environments, these are likely to sorb onto plastic surfaces through direct adsorption, incorporation into carbonate precipitates, or entrapment within biofilms, which create chemically active microenvironments favouring metal binding.^49^ Traces of U showed a similar surface enrichment pattern, and its co-localization with other seawater-derived elements suggests that it was sorbed during environmental exposure, potentially through similar mechanisms. In contrast, elements such as Cd and Sb—though also present in the environment—are commonly used as pigments or flame retardants in plastic manufacturing. In several samples, these elements showed internal intensity gradients, with higher levels in the centre and lower towards the exposed edges. A similar gradient was observed in the colour of the plastic, with more intense coloration preserved in the core and faded or bleached zones near the edges. This alignment suggests that weathering processes may be driving the outward migration of these additives.

The elemental patterns may reflect leaching, where elements are lost from the material into the environment, or diffusion, where elements redistribute internally without being fully dissociated from the plastic matrix. Although the precise mechanisms are uncertain, the co-occurrence of discoloration and elemental depletion supports the idea that long-term environmental exposure alters both the physical appearance and chemical composition of the material. Similar surface-depleted profiles were also observed for Ba and Al, elements typically associated with inorganic fillers rather than pigments. Although not linked to colour change, their gradients indicate that other types of additives may also be affected by environmental exposure. These findings point to broader changes in the internal composition of plastics following prolonged weathering. Some elements, however, did not show one distinct pattern but rather a dual behaviour. For example, Zn and Mg, both presented an internal gradient pattern consistent with a re-distribution of embedded additives, and a surface accumulation suggestive of sorption from seawater or biofilms. This duality illustrates how weathered plastics can both release and sorb metals over time. Overall, these elemental maps, combined with visual observations, suggests that W4 had undergone substantial weathering and supports the idea that increased surface roughness enhanced sorption from seawater. However, the absence of matrix-matched standards and full mass balance assessments limits definitive conclusions about the mechanisms driving these gradients.

To further illustrate these complex spatial patterns, a false-colour RGB composite map was created by overlaying individual elemental distributions (Fig. 5c). This composite visualization provides an overview of the metal distribution across the plastic cross section and highlights areas where elements associated with surface sorption (*e.g.*, Na, Zn) and potential additive loss (*e.g.*, Cd, Zn) are spatially concentrated.

**Fig. 6:**
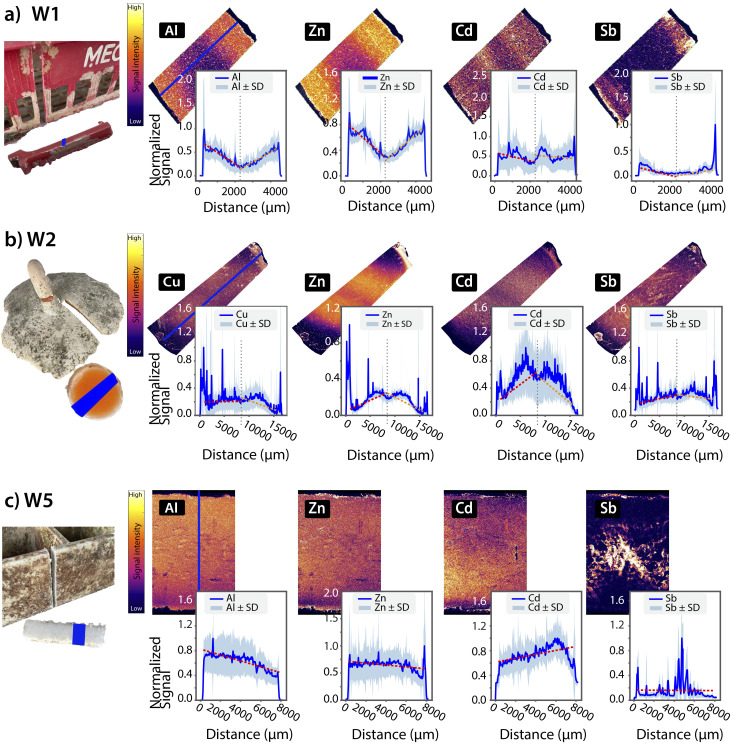
False-colour elemental distribution maps and corresponding cross-sectional intensity profiles (blue) for selected weathered plastic items. Red dashed lines represent segmented linear regression fits applied to the left and right portions of the profile, divided at a local inflection point near the centre (a) and (b), while subplot (c) displays a single linear regression fit across the centre region of the profile. (a) W1: elements such as Al, Zr, Cd, and Sb showed different gradients patterns (b) W2: profiles for Cu, Zn, Cd, and Sb show both higher intensities on the plastic surface (sorption) and internal gradients (leaching). (c) W5: Al and Zn exhibited a one directional gradient (top to bottom), while Cd showed an opposite direction gradient (bottom to top). Similar to W2, Sb displays localized hotspots.

Similar to the pristine items, cross-sectional elemental maps and corresponding intensity profiles were generated for selected weathered samples (Fig. 6). Unlike the pristine plastics, where no consistent gradients were observed, weathered items often showed clear spatial patterns—particularly gradients pointing to the leaching of certain elements. These gradients were evident both in the false-colour elemental maps and in the quantitative profiles, where signal intensities decreased from the core toward the surface, or *vice versa*. Such patterns are indicative of internal migration and release of additives, likely triggered by environmental factors such as UV irradiation, contact with seawater, and mechanical stress. Unlike previous studies focused only on the subsurface,^24^ here our full cross-sectional mapping spans several millimetres—capturing the entire depth of the plastic item, from the weathered edges through the core and back to the weathered edges. This enabled a more comprehensive evaluation of leaching and sorption processes, revealing internal gradients that would have remained undetected with surface-limited techniques.

To further interpret these spatial trends, we examined the distribution profiles of individual elements commonly present as fillers or additives in plastics (Fig. 6). For example, Al, commonly used as a filler, shows a clear concentration gradient from the interior to the surface, suggesting internal redistribution or partial leaching. Cd also displays such gradients. In item W2, visible decolouration is evident (Fig. 4), and this fading may reflect Cd loss from the material. This interpretation is supported by bulk ICP-MS measurements, which revealed elevated Cd concentrations—levels unlikely to result from environmental sorption alone due to low ng L^−1^ concentrations in the surface waters of the NPGP,^50^ and more plausible with Cd being incorporated as a pigment. These findings align with those of Liu *et al.*, who demonstrated that sunlight-mediated Cd release occurs from coloured MPs containing Cd-based pigments in aqueous environments.^51^ Their study highlights that the photo-dissolution of Cd pigments, leads to Cd^2+^ leaching, a process driven by polymer matrix degradation under UV light exposure. Sb, another common plastic additive,^13,52^ exhibited a mix of hotspots, internal gradients and surface-enriched patterns. The observed patterns for Cd and Sb are environmentally relevant due to their known toxicity and potential bioavailability, further accentuating the role of weathered plastics as reservoirs of hazardous trace metals. In all cases, both edges were exposed to seawater. While items W1 and W2 present bidirectional gradients, item W5 displayed a unidirectional gradient suggesting that the object may have been floating mostly upright in the water, resulting in asymmetrical exposure.

To quantitatively assess the bidirectional gradients observed in weathered samples W1 and W2, segmented linear regression fits were applied to the left and right halves of each signal intensity profile. The split point was determined visually, based on where the signal clearly changed direction or flattened out near the middle of the profile. For item W5, the gradient appeared unidirectional and so a single linear regression was fitted to the central region of the signal intensity profile, excluding the edges. The resulting *R*^2^-values, *p*-values, and signal change percentage are summarized in Table 2. These metrics are reported as indicators of relative spatial patterns, rather than absolute chemical changes.

Summary of linear regression statistics for normalized cross-sectional intensity profiles, recorded in weathered plastics from Fig. 6. Directionality is defined as left = exterior edge to core, right = core to opposite exterior edge for items W1 and W2, and top to bottom for W5. *R*^2^-values, *p*-values, and percentage changes in normalized signal intensity reflect relative spatial patterns within each sample, highlighting trends in enrichment or depletion, rather than absolute concentration changes

**Table:** 

	Element	Left segment			Right segment		
		*R* ^2^	*p*	% Change	*R* ^2^	*p*	% Change
W1	Al	0.85	5.3 × 10^−17^	−186	0.92	1.9 × 10^−23^	168
	Zn	0.79	2.4 × 10^−14^	−151	0.91	6.6 × 10^−23^	129
	Cd	0.26	7.6 × 10^−4^	−19	0.06	1.3 × 10^−1^	39
	Sb	0.81	2.3 × 10^−15^	−444	0.65	1.5 × 10^−10^	380
W2	Cu	0.01	2.2 × 10^−1^	27	0.47	7.4 × 10^−21^	−75
	Zn	0.55	1.0 × 10^−26^	60	0.55	7.5 × 10^−26^	−92
	Cd	0.53	6.0 × 10^−25^	43	0.58	3.9 × 10^−28^	−75
	Sb	0.17	3.2 × 10^−7^	37	0.06	4.8 × 10^−3^	−35

**Table:** 

	Element	*R* ^2^	*p*	% Change			
W5	Al	0.48	1.05 × 10^−39^	53.3			
	Zn	0.04	2.02 × 10^−8^	62			
	Cd	0.10	7.74 × 10^−14^	158			
	Sb	0.00	9.57 × 10^−1^	0			

In W1, elements such as Al, Zn, and Sb exhibited statistically significant bidirectional gradients, with opposite slopes but comparable magnitude, suggesting relative enrichment or depletion from both surfaces' edges. Al and Zn showed over 150% change in normalized signal intensity from the core to the edges, highlighting substantial spatial elemental redistribution within the sample. Cd in W1 and Cu in W3 showed more variable behaviour, with lower *R*^2^-values, reflecting imperfect fits to linear models. This discrepancy suggests that while the overall directional trend is real, the signal is affected by localized heterogeneities, non-uniform additive dispersion or weathering artifacts. Some profiles contained pronounced spikes and irregularities that reduced fit quality without negating the presence of relative directional change.

Although many of the fitted slopes were statistically significant (*p* < 0.001), linear regressions may oversimplify the processes driving metals redistribution in weathered plastics. Metal leaching in polymers is often governed by diffusion, which follows non-linear kinetics and can produce exponential or sigmoidal concentration profiles, especially in weathered or aged plastics where surface oxidation, micro-cracks and matrix degradation alter diffusion dynamics.^53,54^ Additionally, mechanisms such as additive migration, photo-oxidative restructuring, or surface contamination could also produce non-linear or spatially variable patterns that are not well captured by linear models. Thus, while the current linear approach provides a useful metric for identifying relative directional trends and comparing spatial patterns across samples, it should not be interpreted as a quantitative measure of leaching or sorption rates.

### 3.4. Comparing elemental distributions in pristine and weathered plastics

**Fig. 7:**
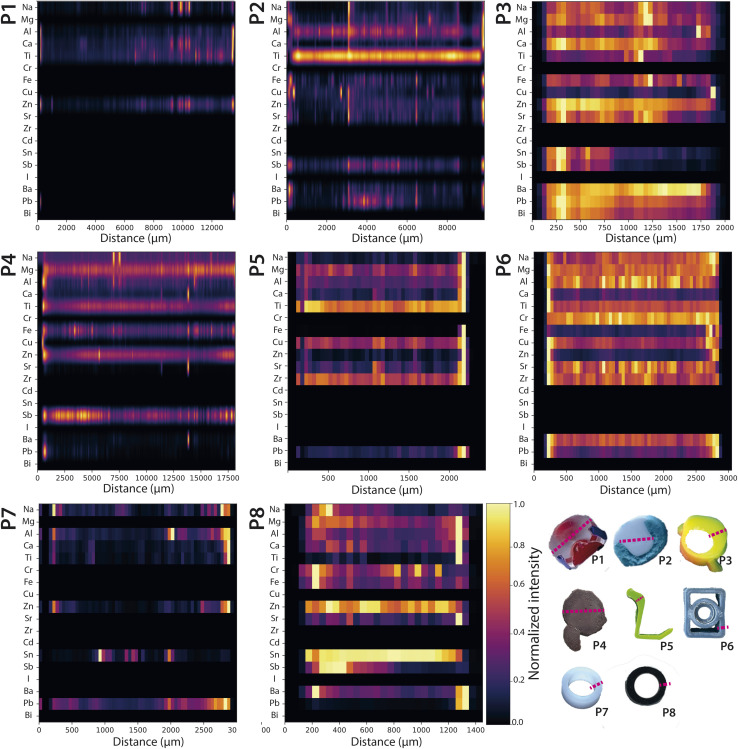
Pristine plastics – normalized signal intensity profiles measured across each sample with a resolution of 5 μm per pixel, visualized as a stacked horizontal heatmaps. The colour gradient represents scaled intensity values (yellow – intense signal, dark – low to no signal). Apparent differences in resolution are visual artefacts due to plotting regions of different length within fixed-width figures; all data were acquired at the same spatial resolution. The largely uniform distributions indicate the absence of leaching or surface sorption.

**Fig. 8:**
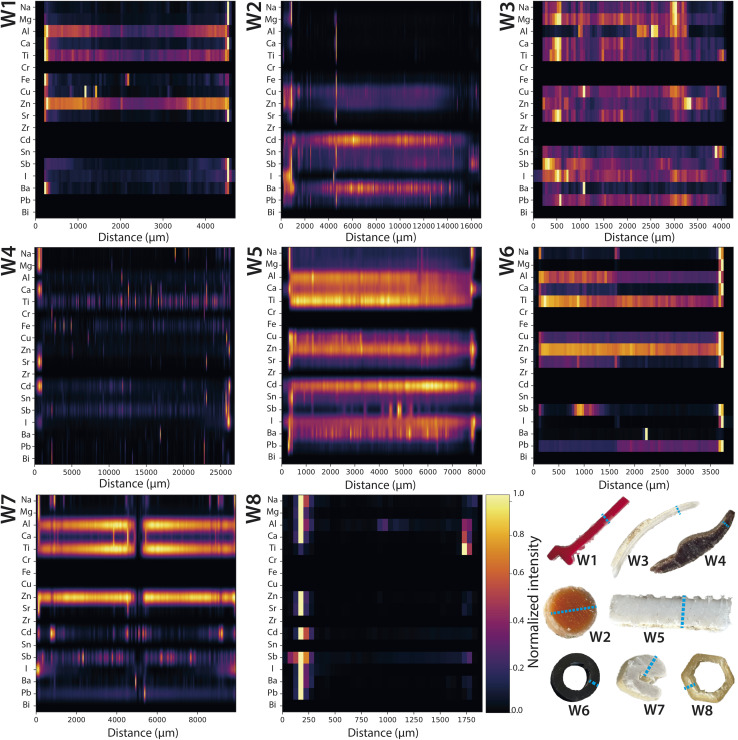
Weathered plastics – normalized signal intensity profiles measured across each sample with a resolution of 5 μm per pixel, visualized as a stacked horizontal heatmaps. The colour gradient represents scaled intensity values (yellow – intense signal, dark – low to no signal). Apparent differences in resolution are visual artefacts due to plotting regions of different length within fixed-width figures; all data were acquired at the same spatial resolution. Observed internal gradients and surface enriched edges indicate relative spatial redistribution of intrinsic elements and potential sorption of seawater constituents, respectively.

To complement the focused examples presented earlier, we normalized the intensity values of a subset of elements and visualized them using heatmaps across both pristine and weathered plastic samples (Fig. 7 and 8). This approach provided a comparative reference and helps visualize broader trends in elemental distribution. Normalization ensured that elements with vastly different absolute intensities could still be compared on the same scale, highlighting regions where elements are present at high or low concentrations. Although the heatmaps may appear to have different resolutions, all areas were scanned with the same resolution of 5 μm per pixel. The discrepancy is a visual representation artifact due to plotting regions of different length within fixed-width plots, compressing or stretching the spatial axes. The resolution of the actual scanned data remains unchanged. The heatmaps allow visualization of the concurrent presence of elements, permitting for the identification of patterns, co-locations, or absence of certain elements in specific regions of the sample. It should be noted that these profiles reflect relative spatial trends within each sample and are not indicative of absolute concentrations.

Similar to the profiles in Fig. 2 and 6, a comparison of Fig. 7 and 8 confirmed that pristine samples generally exhibited uniform elemental distributions without clear concentration gradients. This included additives such as Al, Ti and Zn, which displayed uniform signal intensity profiles across pristine cross-sections in comparison to clear gradients in weathered cross-sections, with higher signal intensity inside the plastics and depletion on surfaces in contact with seawater. In contrast, elements commonly abundant in seawater—such as Na, Mg, Ca, Fe, Zn, and I—were notably enriched at the edges of weathered plastics, reflecting sorption processes during marine weathering absent in pristine samples. Iodine was entirely absent in pristine materials, confirming seawater origin. These surface accumulations likely arise from physical weathering that increases surface roughness and biofilm development, both of which facilitate metal sorption. Sb was detected in both pristine and weathered plastics, consistent with its known use in plastic manufacturing.^13,52^

While Cd was not detected in the pristine plastic samples purchased in 2024 using LA-ICP-TOFMS, very low levels were reported in the bulk analysis using ICP-QQQ-MS. In contrast, weathered samples W2, W4, W5 and W7 showed distinct signal intensity gradients for Cd, which correlated with colour gradients, suggesting its historical use as pigment, as discussed in previous sections. These observations underscore the impact of analytical sensitivity on trace element detection and indicate that Cd is largely absent or present only at trace levels in contemporary plastics, which aligns with current regulations that have largely phased out its use in plastic production due to its toxicity.^13,55^

Lastly, regarding asymmetric weathering, W6 does not show clear gradients but exhibited an abrupt transition, indicative of a bi-composite structure (see Fig. S3). Additionally, the cross-sectional analysis showed that the outer edge exhibited a notably higher concentration of seawater constituents. As this sample is part of a pipe, this suggests that the exterior of the pipe had been exposed more to seawater than the interior, leading to a differential accumulation of elements. Similarly, W8, another hollow object, showed a clear difference in intensity across its surface. Here, the left rim had a higher intensity, which is consistent with the exterior side being more in contact with seawater, further supporting the idea of differential exposure. Lastly, W7, with a prominent crack running along its centre, presents an interesting case where the crack likely served as a pathway for seawater to infiltrate the material. This increased exposure resulted in a higher concentration of seawater constituents along the crack, highlighting the effect of structural damage on the materials interaction with its environment.

### 3.5. Analytical considerations and future standardisation requirements

In this study, two ICP-MS platforms were employed in a complementary manner: bulk ICP-QQQ-MS was used for total metal quantification after acid digestion, and LA-ICP-TOFMS was used for high-resolution, spatially resolved elemental mapping. Not all elements observed in the LA-ICP-TOFMS maps were measured in the bulk ICP-QQQ-MS digests due to the limited set of 49 elements scanned. Conversely, not all elements quantified after sample digestion were observed in the LA-ICP-TOFMS elemental maps. While the focus of this study was to explore spatial elemental trends rather than to perform a quantitative comparison of both analytical platforms, these discrepancies can be explained through several factors.

First, analytical sensitivity differs between the ICP-MS instruments used. ICP-QQQ-MS offers lower detection limits than ICP-TOF-MS (LOQ are presented in Table S5).^29^ Second, the instruments were tuned differently; the quadrupole system was operated in collision/reaction cell mode with O_2_ and He to enhance sensitivity for elements affected by spectral interferences. While this is a useful and widely accepted approach for targeting specific analytes, it can also generate additional reaction products.^56^ In a TOFMS system, which measures the full mass range simultaneously, these unintended species may overlap with analytes of interest, potentially introducing new interferences. To avoid this, the ICP-TOFMS was operated in no-gas mode to preserve full-spectrum clarity and minimize secondary spectral complexity. The impact of these differences in optimization strategies was particularly evident in the case of Se. With ICP-QQQ-MS, the use of O_2_ reaction gas enabled a mass-shift approach, allowing detection of the ^80^Se^16^O^+^ product ion at *m*/*z* 96. This approach took advantage of the high natural abundance of ^80^Se, resulting in improved sensitivity. In contrast, the ICP-TOF-MS could not use mass-shift technique and was therefore limited in no-gas mode to monitoring ^78^Se, a less abundant and inherently less sensitive isotope. Similarly for Cd, pristine samples contained only trace amounts detectable by bulk ICP-QQQ-MS, which remained below the limits of detection of LA-ICP-TOFMS. In contrast, weathered samples exhibited Cd signals detectable by both techniques, with LA-ICP-TOFMS maps revealing relative spatial patterns. These observations highlight that trace elements present at sub-ppm levels may be missed in spatially resolved mapping due to instrument sensitivity. Subsequently, differences in sample introduction between the two systems influenced sensitivity. Solution nebulization generally provides more consistent aerosol transport and ionization compared to laser ablation, which is subject to variables such as ablation efficiency, plasma loading, and elemental fractionation. A final consideration is sample representativeness. For bulk ICP-QQQ-MS a few milligrams of material were digested providing an average elemental composition, whereas LA-ICP-TOFMS maps spanned only a few mm^2^. Consequently, localized variations or trace elements may be missed in small-area mapping, and bulk analyses may be more sensitivite for averaged compositions. These differences underscore the importance of integrating both approaches for a comprehensive assessment of elemental distributions. Overall, these observations emphasize the need for standardized protocols.

Nevertheless, the strength of LA-ICP-TOF-MS lies in its capacity to capture the full mass spectrum at high acquisition rates, enabling simultaneous mapping of numerous elements in a single measurement. This screening capability was particularly useful for detecting seawater-associated elements such as I and Cl, which were not targeted in the ICP-QQQ-MS analysis. Despite its low ionization efficiency, I was consistently detected in the elemental maps, suggesting surface accumulation from the marine environment. Cl was also detected, but it produced high intensity signals and was notched to prevent detector saturation.^29^

While total elemental concentrations in acid digests could be quantified using common multi-element ICP-MS standard solutions, quantification of the elemental maps remained semi-quantitative due to the lack of matrix-matched standards for plastics. While glass-based standards such as NIST SRM 612 were useful for instrument tuning in this study, their ablation behaviour differs substantially from that of polymer matrices and are therefore not ideal for accurate quantification of metals in polymer matrices. Thus, to advance quantitative capabilities in future studies, the use of matrix-matched polymer certified reference materials (CRMs) is essential. In addition to SRM 2858 from NIST, potential candidates for quantification include two PE-based CRMs (ERM-EC680m and ERM-EC681m), a PVC CRM (NMIJ CRM 8123-a), two PP CRMs (NMIJ CRM 8133-a and a Sigma-Aldrich quality control material), as well as an ABS CRM (BAM-H010). These standards, which span multiple common plastic types, have been characterized for both certified and non-certified elemental content,^34^ making them ideal for quantification purposes. Incorporating these in future LA-workflows will improve measurement accuracy and help overcome matrix-related biases, especially when assessing metal distribution and leaching potentials in environmentally aged plastics.

Lastly, to gain a deeper insight into the underlying mechanisms governing metal redistribution in weathered plastics, future studies will require well-defined weathering histories. Experimental designs should incorporate matched pristine and weathered samples exposed to controlled and standardised conditions (*e.g.*, UV radiation, mechanical abrasion, biofouling), in order to isolate weathering effects from initial material heterogeneity. Quantitative assessment of elemental loss or uptake will benefit from mass balance approaches. While linear regression offered a useful starting point for identifying trends, more advanced models accounting for diffusion, sorption dynamics, and matrix degradation are needed to better characterize transport mechanisms in aged plastics.

## 4. Conclusions

This exploratory study demonstrates the potential of LA-ICP-TOFMS for visualizing elemental distributions in weathered marine plastics, offering a new lens to link structural changes to chemical patterns arising from environmental exposure. Similar to trace element zoning observed in geological samples, some of the gradients observed in these plastic cross sections appeared to reflect diffusion-driven redistribution processes. While the mechanisms and materials differ, the elemental patterns—localized enrichments (sorption), depletion zones (leaching), and sharp transitions (bi-composite material)—underscore the broader applicability of spatially resolved elemental mapping. This capability allows to construct detailed cross-sectional maps of elemental distributions in weathered plastics. The elemental maps presented here showcase visual correlations between physical degradation (*e.g.*, cracking, roughening) and metal distributions. To support these visual interpretations, normalized linear regression analyses were applied to intensity profiles across the plastic cross sections, confirming the presence of spatial gradients of multiple elements and reinforcing the interpretation of leaching and sorption. For example, pigment elements such as Cd exhibit clear intensity gradients suggestive of leaching, while seawater-derived elements such as Na, Mg, and I, are concentrated along weathered surfaces and appear to infiltrate the plastic matrix through structural defects—implying that sorption is facilitated through weathering. Importantly, this study focused on large plastic items naturally weathered in the ocean—offering insights into how environmental exposure to sunlight, seawater, and mechanical stress can drive crack mediated infiltration and additive redistribution within plastic matrices. While the plastic items analysed during this survey presents some unknowns including initial compositions and exposure durations, the presented approach would lend itself to be used for more controlled, systematic studies of plastics weathering to assess the extent and rate of leached and sorbed metals in various environmental contexts. Finally, the development and use of matrix-matched polymer reference materials will be essential for enabling quantitative assessments and advancing our understanding of metal redistributions in plastics.

## Author contributions

L. H. performed conceptualization, project administration, resources, methodology, investigation, validation, formal analysis, data curation, visualization, writing – original draft and writing – review & editing, and response to reviewers. M. E. contributed with conceptualization, providing resources, and with writing – review & editing. D. M. M. supported with conceptualization and writing – review & editing. All authors have reviewed and approved the final version of the manuscript.

## Conflicts of interest

M. E. is employed by The Ocean Cleanup, a non-profit organization aimed at advancing scientific understanding and developing solutions to rid the ocean of plastic, headquartered in Rotterdam.

## Supplementary Material

JA-040-D5JA00223K-s001

## Data Availability

The data that support the findings of this study are available from the corresponding author upon reasonable request. Supplementary information: an overview of the analzyed plastics items – weathered and pristine, operating conditions for ICP-MS and LA-ICP-TOFMS, FTIR characterization results, and bulk digestion results. See DOI: https://doi.org/10.1039/d5ja00223k.
