# Supplementary material for: Investigating metal distribution patterns in pristine and ocean-weathered plastics using LA-ICP-TOFMS
Source: J Anal At Spectrom. 2025 Sep 8;40(10):2841–56. doi: 10.1039/d5ja00223k (PMC12415543; doi:10.1039/d5ja00223k)

Supplementary Information (SI) for JAAS.
This journal is © The Royal Society of Chemistry 2025

Supporting Information -

Investigating metal distribution patterns in pristine and ocean-weathered plastics using LA-ICP-TOFMS

Lyndsey Hendriks[1], Matthias Egger[2,3], & Denise M. Mitrano[4]

[1] Institute of Analytical Chemistry, University of Vienna, Vienna, Austria
[2]The Ocean Cleanup, Coolsingel 6, 3011 AD Rotterdam, The Netherlands
[3]Empaqtify, Ullmannstrasse 13a, 9014 St. Gallen, Switzerland
[4]Department of Environmental Systems Science, ETH Zurich, Universitätstrasse 16, 8092 Zurich, Switzerland

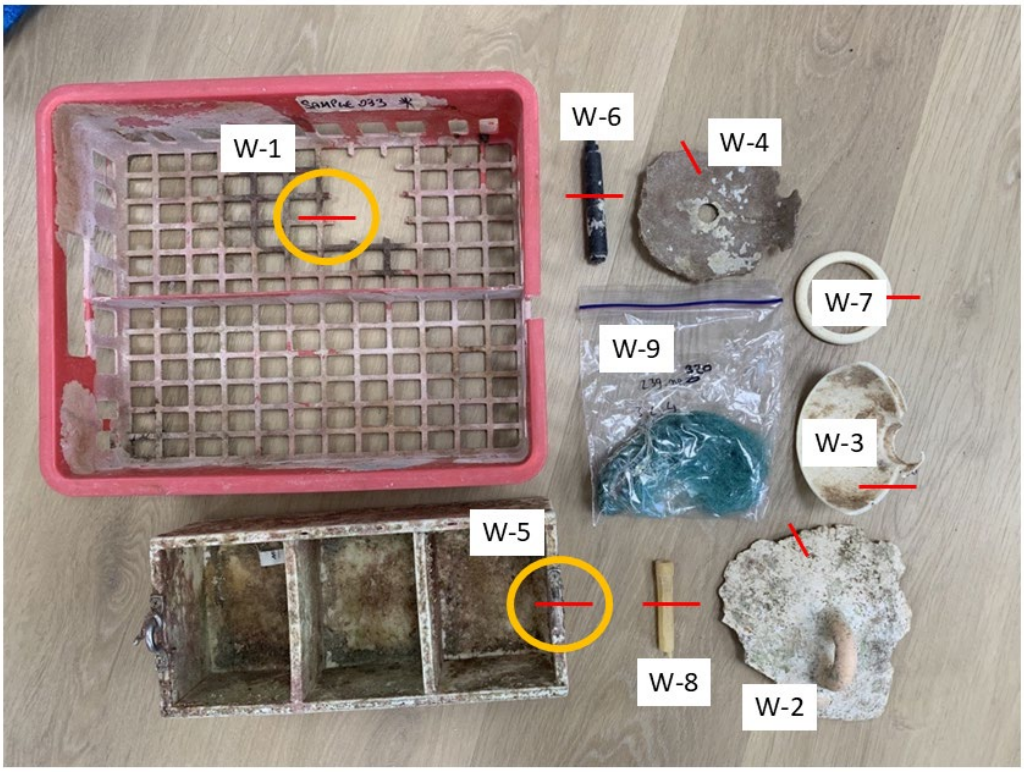

Figure S1: Overview of the collected plastic samples retrieved from the North Pacific Garbage Patch, a plastic accumulation area located between Hawaii and California. Samples W1 to W8 were analysed by LA-ICP-TOFMS. W9 was a fishing net and was not analysed, as it was not possible to get a cross section of the fibres.

Table S1: Overview of the pristine plastic samples (P1-P8) and the weathered plastic samples (W1-W8). The ablated area is highlighted in blue and overlayed on the cross-sections. The ablated area is estimated based on an equivalent rectangular shape (width (µm) x height (µm)).

Name	Overview photo and cut	Cross section and ablated area	Name	Overview photo and cut	Cross section and ablated area
P1	Natural toothbrush 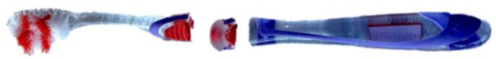	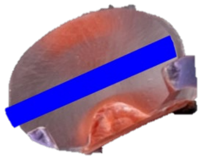 12850 x 6880	P5	Green plastic handle 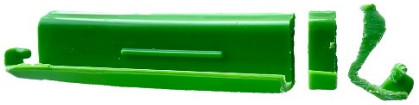	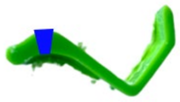 2630 x 2440
P2	White toothbrush 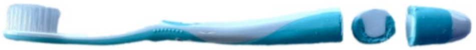	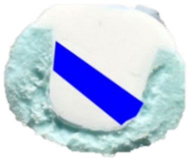 7820 x 8430	P6	Blue plastic handle 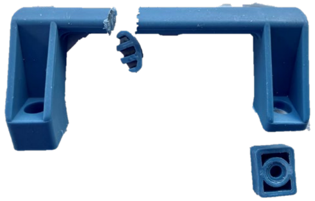	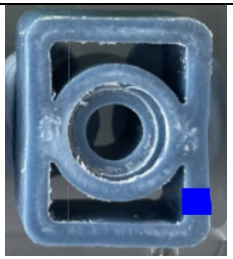 3130 x 2800
P3	Colourful fishing vertical bouncer 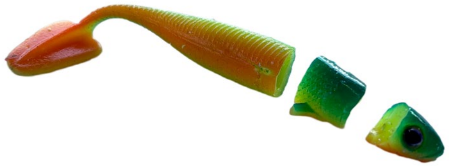	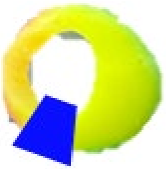 2310 x 3110	P7	PTFE tubing 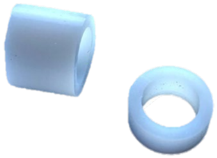	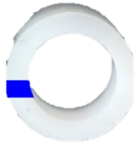 1870 x 3410
P4	Dinosaur toy 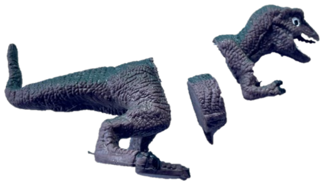	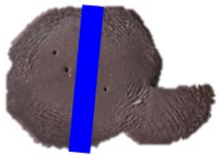 2760 x 18410	P8	TEFLON tubing 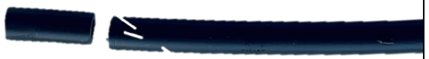	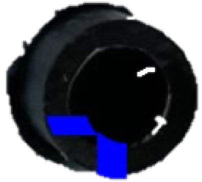 1080 x 1680

Name	Overview photo and cut	Cross section and ablated area	Name	Overview photo and cut	Cross section and ablated area
W1	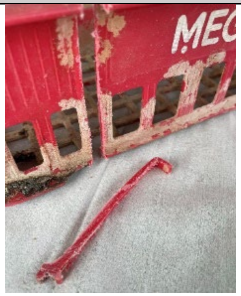 Magenta basket	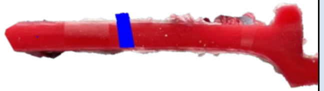 5100 x 3230	W5	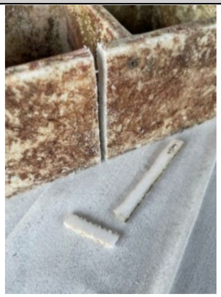 White box	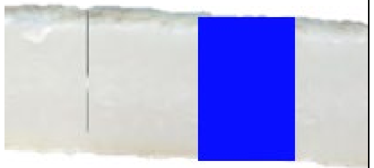 5540 x 8525
W2	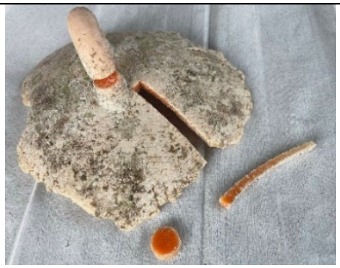 Orange buoy	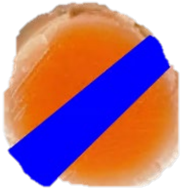 14540 x 13650	W6	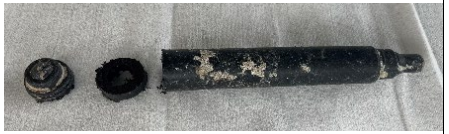 Black pipe	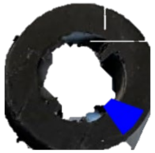 4620 x 4110
W3	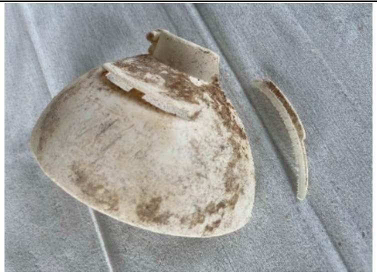 White buoy	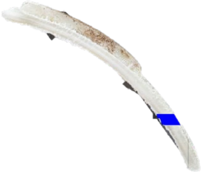 6250 x 2845	W7	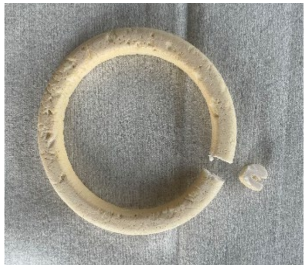 Ring	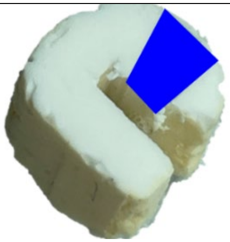 4910 x 6105
W4	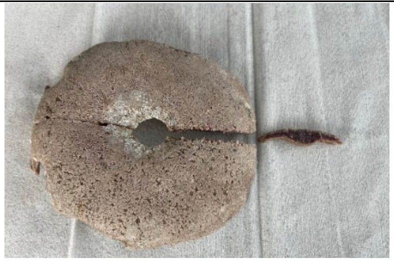 Brown buoy	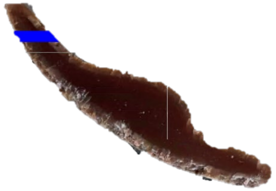 6930 x 1635	W8	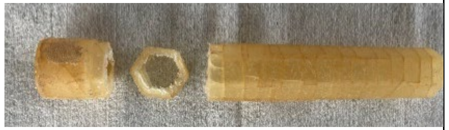 Yellow pipe	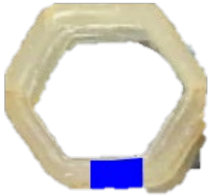 3360 x 2035

Table S2: Operating conditions for ICP-MS

ICP-MS parameters	Value
Sample introduction	MicroMist nebulizer
Nebulizer gas [L min^{-1}]	1.1
Sampling depth [mm]	5
Plasma power [W]	1550
Auxiliary flow [L min^{-1}]	0.8
Cool Flow [L min^{-1}]	15
Cell gas flow O_2 [L min^{-1}]	0.32
Cell gas flow H_2 [L min^{-1}]	4

Table S3: Operating conditions for LA-ICP-TOFMS

Laser Parameters	Value	ICP-TOFMS parameters	Value
Laser energy density [J cm^{-2}]	1-3*	Injector	DCI one piece
Repetition rate [Hz]	100	Nebulizer gas [L min^{-1}]	0.9
Scan rate [µm s^{-1}]	500	Sampling depth [mm]	5
Spot size [µm]	10 square	Plasma power [W]	1550
Interspacing in x and y [µm]	5	Auxiliary flow [L min^{-1}]	0.8
He carrier gas flow rate [mL min^{-1}]	320	Cool Flow [L min^{-1}]	14
Sample flow rate [mL min-1]	280		

*Depending on ablation behaviour

Table S4: FTIR characterization results

To identify common polymers using IR spectroscopy, specific vibrational bands characteristic of their functional groups can be used as molecular fingerprints. The table below highlights the most prominent bands for five widely used polymers.

Item	Characteristic bands observed	Polymer type
P1	C=O (ester carbonyl): ~1715 cm^{-1}, aromatic C–H bending: ~870 cm^{-1}, C–O–C (ether): ~1240 cm^{-1}	PET
P2	C–H stretch (methyl): ~2950, ~2915, ~2838 cm^{-1}, CH_3 bending: ~1375 cm^{-1}, CH_2 bending: ~1455 cm^{-1}	PP
P3	aliphatic C–H groups: ~2960–2874 cm^{-1}, C=0 (ester carbonyl): ~1722 cm^{-1}, C-O stretches; ~1265-1069 cm^{-1}, aromatic C–H bending: ~741, ~651 cm^{-1}	Diisodecyl Phtalate
P4	Aromatic C–H stretch: ~3026 cm^{-1}, Aromatic C=C stretch: 1601, 1493 cm^{-1}, C–H in-plane bend: ~1452 cm^{-1}, C–H out-of-plane bend: 750–690 cm^{-1}	PS
P5	C–H stretch (methyl): ~2950, ~2915, ~2838 cm^{-1}, CH_3 bending: ~1375 cm^{-1}, CH_2 bending: ~1455 cm^{-1}	PP
P6	C–H stretch (methyl): ~2950, ~2915, ~2838 cm^{-1}, CH_3 bending: ~1375 cm^{-1}, CH_2 bending: ~1455 cm^{-1}	PP
P7	C–F stretch: ~1100–1300 cm^{-1} → very strong, unique to fluoropolymers, Skeletal vibrations: ~630–540 cm^{-1}<br>⚠No C–H bands, unlike hydrocarbon polymers	PTFE
P8	C–F stretch: ~1100–1300 cm^{-1} → very strong, unique to fluoropolymers, Skeletal vibrations: ~630–540 cm^{-1}<br>⚠No C–H bands, unlike hydrocarbon polymers	PTFE
W1	C–H stretch (methyl): ~2950, ~2915, ~2838 cm^{-1}, CH_3 bending: ~1375 cm^{-1}, CH_2 bending: ~1455 cm^{-1}	PP
W2	C–H stretch (CH_2): ~2915, ~2849 cm^{-1}, CH_2 bending: ~1465 cm^{-1}, CH_2 rocking: ~720 cm^{-1}	PE
W3	C–H stretch (methyl): ~2950, ~2915, ~2838 cm^{-1}, CH_3 bending: ~1375 cm^{-1}, CH_2 bending: ~1455 cm^{-1}	PP
W4	C–H stretch (CH_2): ~2915, ~2849 cm^{-1}, CH_2 bending: ~1465 cm^{-1}, CH_2 rocking: ~720 cm^{-1}	PE
W5	C–H stretch (CH_2): ~2915, ~2849 cm^{-1}, CH_2 bending: ~1465 cm^{-1}, CH_2 rocking: ~720 cm^{-1}	PE
W6	C–H stretch (methyl): ~2950, ~2915, ~2838 cm^{-1}, CH_3 bending: ~1375 cm^{-1}, CH_2 bending: ~1455 cm^{-1}	PP
W7	C–H stretch (CH_2): ~2915, ~2849 cm^{-1}, CH_2 bending: ~1465 cm^{-1}, CH_2 rocking: ~720 cm^{-1}	PE
W8	C–H stretch (CH_2): ~2915, ~2849 cm^{-1}, CH_2 bending: ~1465 cm^{-1}, CH_2 rocking: ~720 cm^{-1}	PE

* *PET (polyethylene terephthalate), PP (polypropylene), PS (polystyrene), PTFE (polytetrafluoroethylene), PE (polyethylene)*

<u>References</u>:

- Hummel, D.O. *Infrared and Raman Spectroscopy of Polymers*. Hanser Publishers (2002).
- Chércoles Asensio, R., et al. *Analytical characterization of polymers used in conservation and restoration by ATR-FTIR spectroscopy*. Anal Bioanal Chem 395, 2081–2096 (2009). https://doi.org/10.1007/s00216-009-3201-2

Figure S2: Overview of the metal content measured in the plastic items after acid digestion. Note that items P4, P7 and P8 were not digested nor analysed, and are thus not presented herein. Values below the LOQ were not plotted.

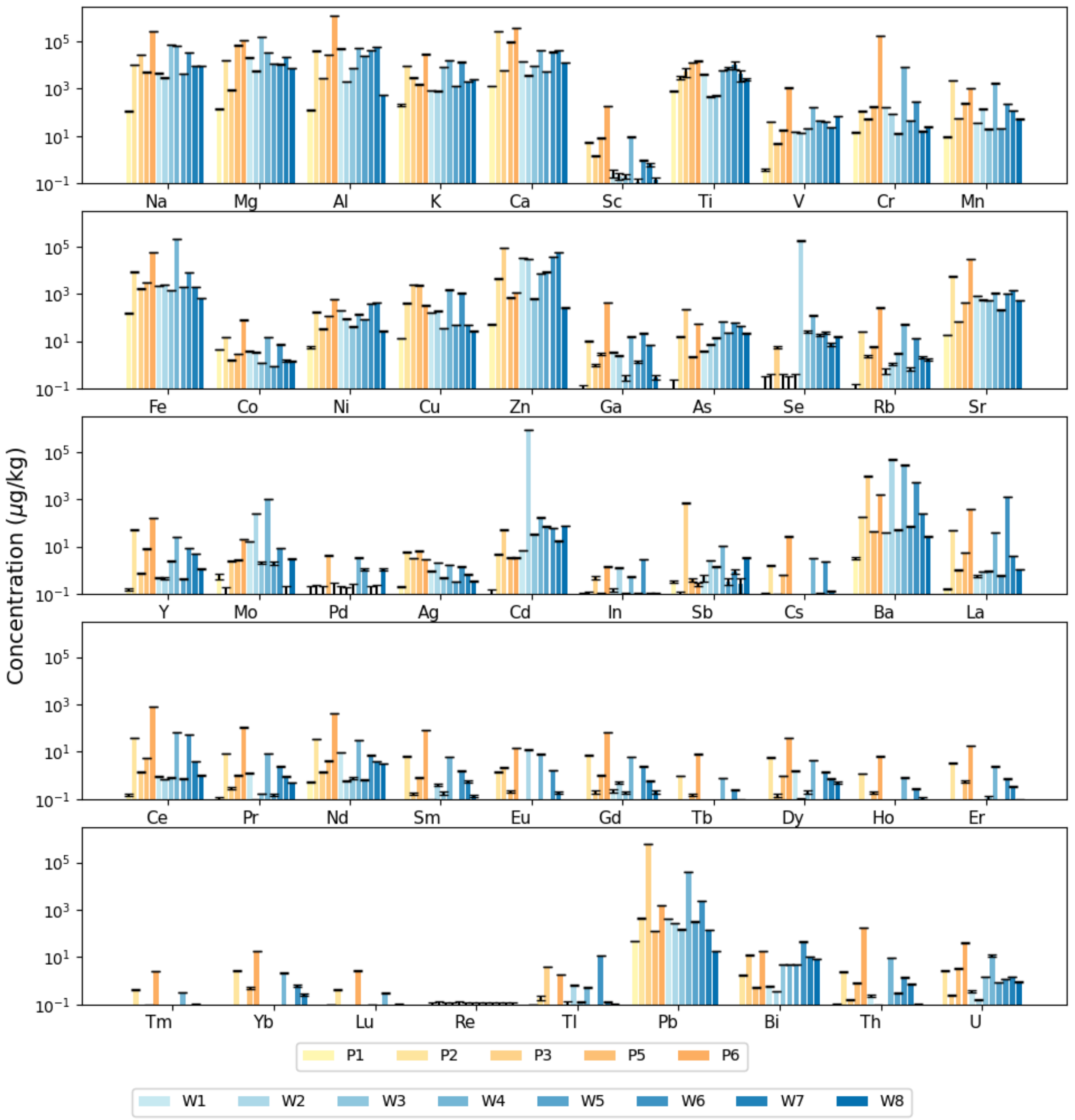

Table S5: Summary of the recorded LOQs. Different elements were measured in He mode to reduce matrix-induced polyatomic interferences (e.g., ArO^+, ClO^+, ArC^+), common in environmental and plastic digestion matrices. While this may slightly reduce absolute sensitivity for some isotopes, it improves quantification accuracy and consistency, especially for metals like V, Cr, Fe, and As that are prone to interference.

Element	*Isotope monitored*	*Mode*	*LOQ (ug/kg)*	*Element*	*Isotope monitored*	*Mode*	*LOQ (ug/kg)*
Na	^{23}Na	He KED	1.47	In	^{115}In	No Gas	0.005
Mg	^{24}Mg	He KED	0.41	Sb	^{121}Sb	No Gas	0.009
Al	^{27}Al	He KED	1.84	Cs	^{133}Cs	No Gas	0.001
K	^{39}K	STD	5.79	Ba	^{137}Ba	No Gas	0.052
Ca	^{43}Ca	He KED	4.50	La	^{139}La	No Gas	0.002
Sc	^{45}Sc	He KED	0.022	Ce	^{140}Ce	No Gas	0.002
Ti	$^{47}Ti \rightarrow {}^{47}Ti^{16}O$	O_2	0.47	Pr	^{141}Pr	No Gas	0.001
V	^{51}V	He KED	0.012	Nd	^{146}Nd	No Gas	0.003
Cr	^{53}Cr	He KED	0.37	Sm	^{147}Sm	No Gas	0.003
Mn	^{55}Mn	He KED	0.030	Eu	^{153}Eu	No Gas	0.001

Fe	^{56}Fe	He KED	0.16	Gd	^{157}Gd	No Gas	0.002
Co	^{59}Co	He KED	0.007	Tb	^{159}Tb	No Gas	0.001
Ni	^{60}Ni	He KED	0.37	Dy	^{163}Dy	No Gas	0.001
Cu	^{63}Cu	He KED	0.068	Ho	^{165}Ho	No Gas	0.001
Zn	^{66}Zn	He KED	0.15	Er	^{166}Er	No Gas	0.002
Ga	^{71}Ga	He KED	0.009	Tm	^{169}Tm	No Gas	0.001
As	^{75}As	He KED	0.068	Yb	^{172}Yb	No Gas	0.001
Se	^{80}Se → ^{80}Se^{16}O	O_2	0.19	Lu	^{175}Lu	No Gas	0.001
Rb	^{85}Rb	He KED	0.029	Re	^{185}Re	No Gas	0.015
Sr	^{88}Sr	He KED	0.053	Tl	Tl	No Gas	0.001
Y	^{89}Y	He KED	0.001	Pb	^{208}Pb	No Gas	0.017
Mo	^{98}Mo	He KED	0.079	Bi	^{209}Bi	No Gas	0.001
Pd	^{106}Pd → ^{106}Pd^{16}O	O_2	0.092	Th	^{232}Th	No Gas	0.005
Ag	^{107}Ag	No Gas	0.009	U	^{238}U	No Gas	0.003
Cd	^{111}Cd	No Gas	0.023				

Table S6: Mechanical properties and visual assessment

The weathered sample clearly exhibits changes in both colour and surface morphology. A distinct gradient from a lighter, more degraded outer layer to a darker inner core is observed in some weathered plastics, likely from UV exposure and interaction with surrounding elements. Additionally, the weathered plastic appears more brittle and fragmented, with rougher and more irregular edges, compared to the smoother surface of the pristine item. Evidence of biofouling is also apparent, with visible growth of microorganisms such as algae and bacteria on the plastic surface. These physical changes align with indicators of environmental aging, such as surface embrittlement, colour alteration, and microcrack formation, all of which can influence the material's sorptive behaviour and leaching properties.

Item	Mechanical properties	Colour	Structure
P1	Hard	Uniform	smooth
P2	Hard	Uniform	smooth
P3	Souple	Uniform	smooth
P4	Souple	Uniform	smooth
P5	Hard	Uniform	smooth
P6	Hard	Uniform	smooth
P7	Hard	Uniform	smooth
P-8	Hard	Uniform	smooth
W1	hard	Uniform	Smooth with growth of microorganisms
W2	Brittle	Intense colour gradient	Cracked surface with growth of microorganisms
W3	hard	Fade colour gradient	Cracked surface with growth of microorganisms
W4	Brittle	Intense gradient	Cracked surface with growth of microorganisms
W5	hard	Fade colour gradient	Cracked surface with growth of microorganisms
W6	hard	Uniform	Smooth with growth of microorganisms
W7	hard	Fade colour gradient	Cracked surface
W8	hard	Fade colour gradient	Cracked surface

Figure S3: Elemental maps and corresponding profiles of item W6. Clear distributions are observed as opposed to gradients; similarly abrupt edges are recognized in the profiles. While the sharp transitions indicated a potential bicomposite material, sorption through surface accumulation as well as leaching (Sb) are both also observed.

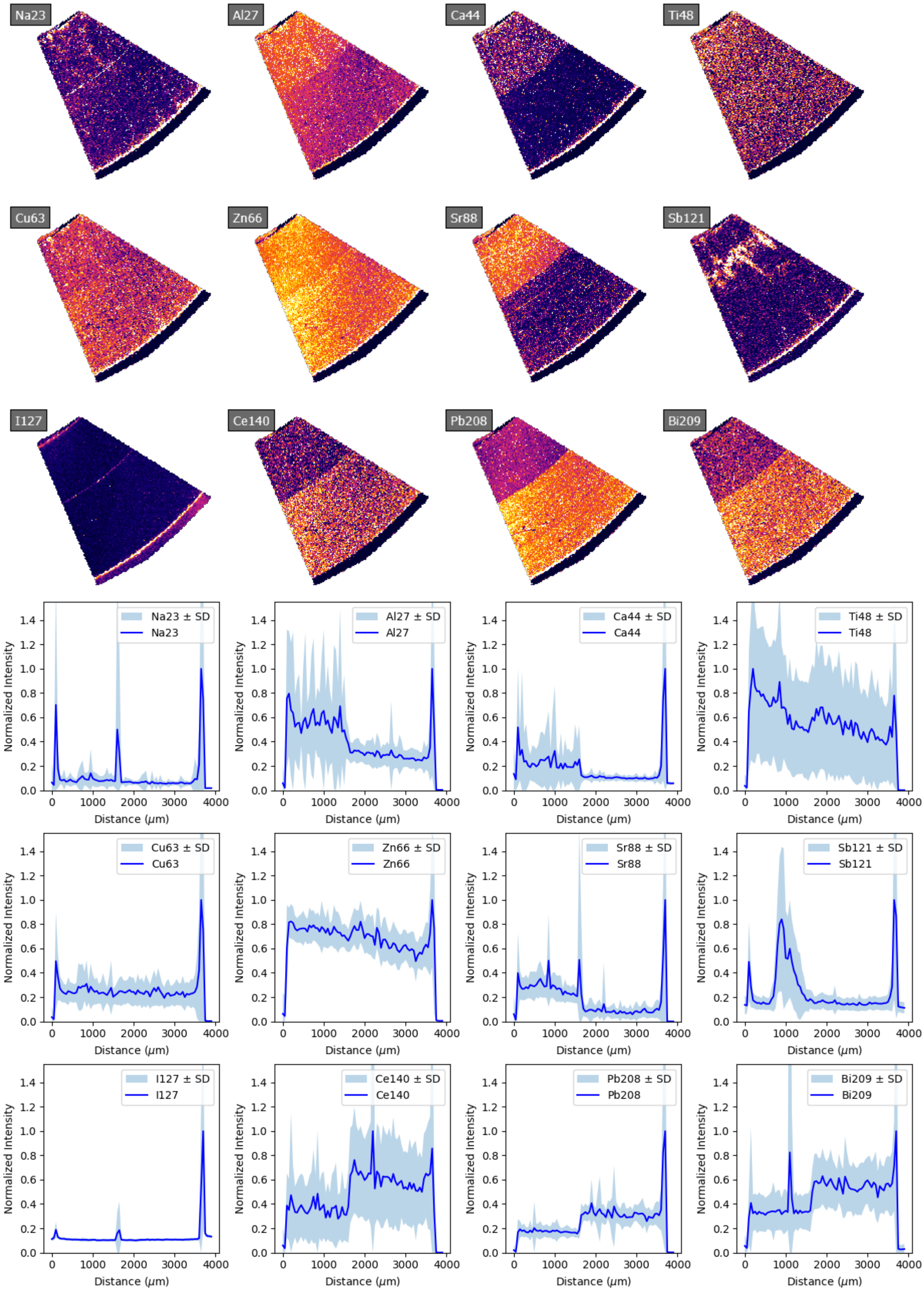

Supplement: JA-040-D5JA00223K-s001 [file JA-040-D5JA00223K-s001.pdf]
